# Effectiveness of a voice-guided tap-and-talk educational tool in enhancing cardiovascular knowledge and self-management in aged patients following percutaneous coronary intervention

**DOI:** 10.1186/s13019-026-04546-3

**Published:** 2026-07-22

**Authors:** Tingting Sun, Lili Liu, Rui Ye, Yaqi Wei, Xia Li

**Affiliations:** 1 Department of Cardiology, Beijing Luhe Hospital, Capital Medical University, Beijing, 101100 China; 2 Department of Geriatrics, Beijing Luhe Hospital, Capital Medical University, Beijing, 101100 China; 3 Department of Nursing, Beijing Luhe Hospital, Capital Medical University, No. 82 Xinhua South Road, Tongzhou District, Beijing, 101100 China

**Keywords:** Coronary heart disease, Aged, Health education, Percutaneous coronary intervention, Self-management

## Abstract

**Objective:**

This study aimed to develop a voice-guided, tap-and-talk educational tool and assess its effectiveness in improving disease-related knowledge and self-management behaviors among older adults following percutaneous coronary intervention (PCI).

**Methods:**

A voice-guided tap-and-talk educational tool was designed to address the specific needs of older adults post-PCI. A cluster randomized controlled trial was conducted in cardiovascular wards. The control group received conventional health education using printed manuals, whereas the intervention group received education via the tap-and-talk interactive tool. Outcomes included the level of knowledge regarding coronary heart disease and engagement in self-management behaviors. Chi-squared test and independent-sample t-test, chi-square test and paired t-test, and univariate repeated-measures analysis of variance (ANOVA) were conducted accordingly.

**Results:**

A total of 96 patients completed the study, including 49 in the experimental group and 47 in the control group.No statistically significant differences were observed in baseline characteristics between the groups (*p* > 0.05). A significant group × time interaction effect was detected for coronary heart disease (CHD)-related knowledge (F = 9.026, *P* < 0.001). At 1 month, 3 months, and 6 months post-intervention, the intervention group demonstrated significantly higher self-management behavior scores than the control group (F = 11.607, *P* < 0.001). Additionally, CHD-related knowledge scores showed a mild downward trend over time in both groups (F = 124.067, *P* < 0.001). Consistent with the interaction effect mentioned above, the intervention group achieved significantly higher CHD-related knowledge scores than the control group at all three follow-up time points (1 month, 3 months, and 6 months post-intervention) (F = 195.188, *P* < 0.001). Scores in the intervention group remained consistently higher across all domains. The tool received high acceptance ratings from participants (mean score 96.04 ± 2.61).

**Conclusion:**

The tap-and-talk voice-guided educational tool was effective in enhancing knowledge and promoting self-management behaviors among older adults following PCI. The tool demonstrated a high level of user acceptability within the target population.

**Supplementary Information:**

The online version contains supplementary material available at https://doi.org/10.1186/s13019-026-04546-3.

## Introduction

The 2025 Global Burden of Disease report identified a sustained a global increase in both the prevalence and mortality associated with cardiovascular disease (CVD) over the past three decades, with ischemic heart disease remaining the leading cause of cardiovascular-related deaths [1]. In China, CVD represents the primary cause of mortality in both urban and rural populations [2]. Reducing cardiovascular mortality requires active promotion and consistent monitoring of cardiovascular health indicators [2]. Percutaneous coronary intervention (PCI) constitutes a primary therapeutic approach for coronary heart disease (CHD), involving the placement of coronary stents to restore perfusion in stenotic or occluded coronary arteries [2, 3]. This procedure alleviates myocardial ischemia and hypoxia, mitigates symptoms, and contributes to improved quality of life [3]. However, long-term outcomes are dependent on the effective implementation of secondary prevention strategies such as pharmacological therapy following PCI, lifestyle modification, and structured postoperative rehabilitation. Effective health education is essential to facilitate the successful implementation of these secondary prevention measures for CHD [4, 5]. 

According to the definition of the World Health Organization (WHO), people aged 60 and above are defined as older adults [6]. Older adults represent the a substantial proportion of individuals undergoing PCI. Age-related cognitive decline, manifesting as impairments in memory, calculation, verbal communication, and spatial reasoning, can negatively impact the ability to comprehend and utilize conventional text-based health education materials [7–9]. These deficits hinder the ability to comprehend and apply traditional text-based educational materials, leading to insufficient understanding of post-PCI care and reduced effectiveness of health education. Consequently, adherence to treatment and the implementation of secondary prevention strategies may be compromised [10, 11]. To improve knowledge acquisition and adherence among older adults following PCI, educational tools must be tailored to their cognitive and perceptual capabilities. An effective tool should offer intuitive operation, engaging and easily comprehensible content, and utilize multisensory delivery with opportunities for repeated exposure to instructional material. Such an approach may improve knowledge retention, support self-management, and promote positive behavioral change.

Cardiac rehabilitation is an evidence-based cornerstone of secondary prevention for post-PCI patients, as recommended by clinical guidelines, and its efficacy in reducing cardiovascular adverse events and improving long-term prognosis has been well established [4]. However, despite its proven value, cardiac rehabilitation remains severely underutilized in China—multicenter surveys have identified low participation rates among post-PCI patients, attributed to barriers such as insufficient awareness, uneven distribution of rehabilitation resources, and limited accessibility for elderly populations [11, 12]. Patient education delivered via mobile applications, group sessions and online courses is less popular among older adults. Elderly patients prefer written materials obtained from medical professionals or downloaded online, yet such materials are often limited by issues including font size and visually aided markers. Well-designed audio-visual resources are also regarded as valuable alternatives [13]. Video content relies on smartphones for playback, whereas audio materials need only basic tools. This critical gap between the clinical need for effective secondary prevention and the underuse of existing cardiac rehabilitation programs underscores the urgency of developing accessible, user-friendly educational interventions tailored to older adults’ cognitive and perceptual characteristics, thereby justifying the development and evaluation of our voice-guided tap-and-talk tool.

In response to these needs, a tap-and-talk voice-guided educational tool was conceptualized and developed. The tool was refined through iterative expert consultation, content modifications, and clinical trials. The finalized application delivers health education content in audio and video formats, presenting data in an accessible, user-friendly manner, enabling users to repeatedly access information by tapping to initiate playback. It functions as an audiobook, with voice narration and integrated code-scanning capabilities. Clinical validation demonstrated favorable user acceptance, ease of use, and stable code-scanning performance. This randomized controlled trial (RCT) was conducted to evaluate the effectiveness of the tap-and-talk educational tool in enhancing knowledge and self-management behaviors among older adults following PCI. No previous publications specifically describing this study-specific tap-and-talk educational package were identified; therefore, its development process, pilot testing, and clinical application are described in detail below to support reproducibility.

## Materials and methods

### Participants

Older adult patients who had undergone PCI were recruited through convenience sampling from the Department of Cardiology, Beijing XX Hospital, between March 2019 and March 2021.

The inclusion criteria were as follows: (1) age ≥ 60 years; (2) absence of visual or auditory impairments, no history of psychiatric disorders, preserved consciousness, intact cognitive function (normal MMSE score), and ability to cooperate with the study; (3) voluntary participation with provision of signed informed consent.

The exclusion criteria were as follows: (1) history of PCI within the preceding 12 months; (2) inability to complete postoperative follow-up due to any limiting factors.

### Sample size calculation

The sample size calculation formula for comparing two independent sample means n = [2(u_α_ + u_β_)σ/δ]^2^ was adopted. With α = 0.05 and β = 0.1, the critical values from the standard normal distribution table were Z_0.05/2_=1.96 and Z_0.1_=1.28. Based on the pilot study results, the mean of the intervention group (*x*ˉ1) was 86.96, the mean of the control group (*x*ˉ2) was 81.06, the standard deviation of the intervention group (s₁) was 8.65, and the standard deviation of the control group (s₂) was 7.73. These values were substituted into the formula to calculate the pooled standard deviation (σ = 8.20) and the mean difference (δ = 5.9). Further substitution into the sample size formula yielded a result of 40.65, which was rounded up to 41. Considering a 20% attrition rate for each group, the final sample size per group was 49.2, rounded up to 50 participants.

### Study methods

Before the experiment commenced, all investigators received a 1-hour training session covering the project overview, data collection methods, and precautions. Health education nurses completed a 30-minute training and assessment on tool operation to ensure they could proficiently train the study participants.

#### Group Allocation

**Fig. 1:**
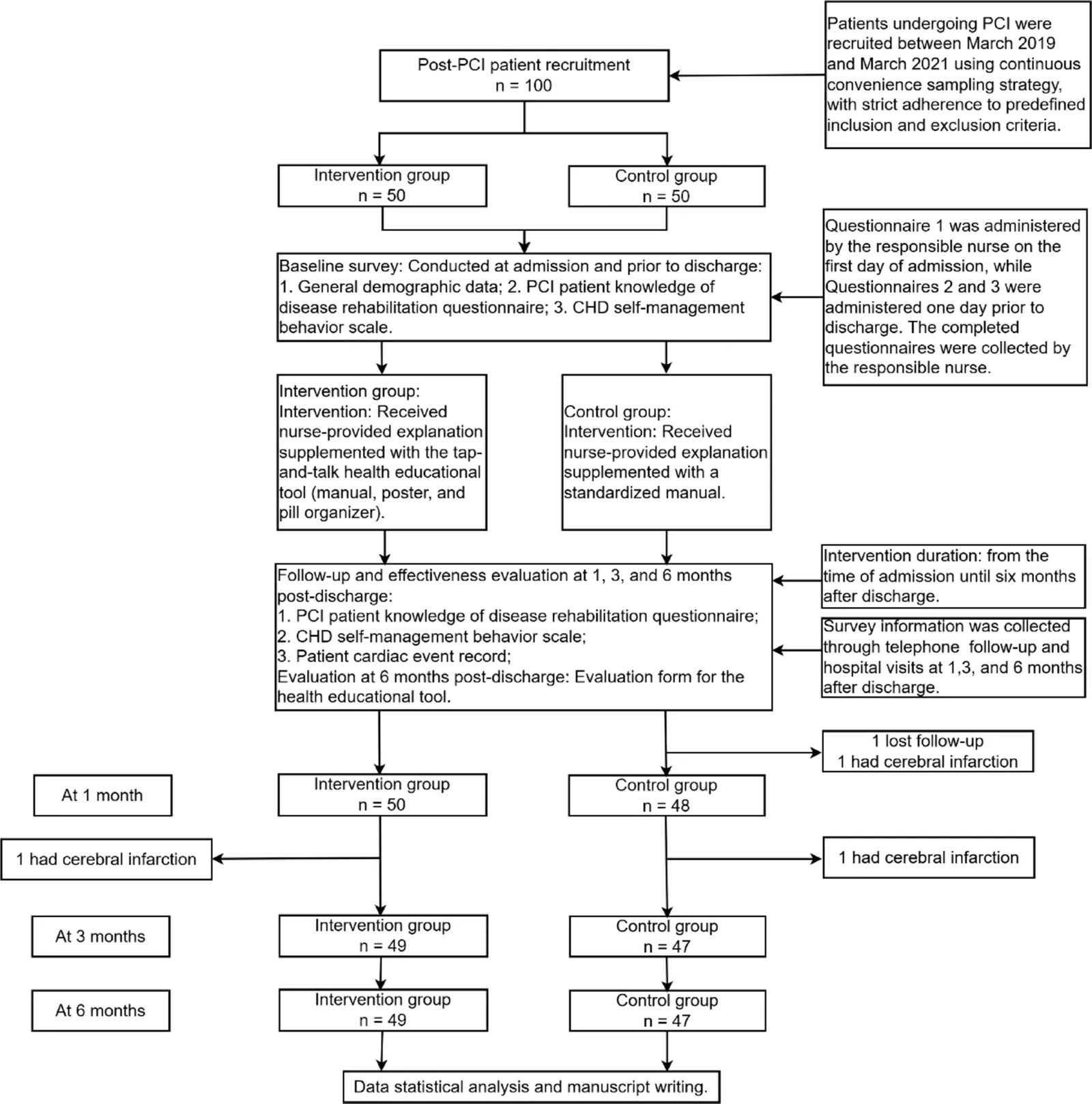
Study flow chart

This study adopted the cluster randomization method, whereby 12 cardiology wards were randomly assigned to either the intervention group (tap-and-talk intervention) or the control group via drawing lots. Patients who met the eligibility criteria were allocated based on ward assignment. Those in the intervention group received the tap-and-talk health educational tool, while those in the control group were provided with a traditional health education manual. The technical roadmap of the intervention is illustrated in Fig. 1).

#### Intervention procedures

##### Control group

Participants in the control group received conventional health education delivered using a standardized manual containing four sections: an overview of coronary artery disease and interventional procedures, clinical manifestations of the disease, essential perioperative care for coronary artery intervention, and health guidance for the six-month period post-discharge. At the time of admission, instruction was delivered by a health education nurse in accordance with the manual. Upon discharge, participants were given a copy of the manual for home use. Weekly text message reminders were sent to encourage review and adherence to the educational material. The intervention period spanned from admission to six months post-discharge.

##### Intervention group

The tap-and-talk health educational tool was developed by a multidisciplinary team comprising of researchers, two PCI clinicians, two nurse specialists (each with over 10 years of clinical experience), and a nursing research expert. The tool was refined through multiple rounds of expert consultation, pilot testing, and content revision prior to finalization for clinical implementation.

The tool used in this study consisted of a commercially available standardized tap-and-talk pen and study-specific educational materials independently developed by the research team. The pen was a mature commercial hardware device that supported customized audio input, point-code binding, and offline voice playback. The educational content was not a manufacturer-provided package. Instead, the research team developed four core modules: basic knowledge of CHD and PCI, typical clinical manifestations of CHD, perioperative nursing points for PCI, and six-month post-discharge rehabilitation and self-management guidance.

The educational content was developed through a structured process. First, the research team reviewed relevant clinical guidelines and post-PCI care requirements and drafted standardized educational texts for the manual, wall poster, and medication-related reminders. Second, the materials were reviewed and revised by a multidisciplinary team consisting of researchers, two PCI clinicians, two cardiovascular nurse specialists with more than 10 years of clinical experience, and one nursing research expert to ensure clinical accuracy, readability, and suitability for older adults. Third, a senior cardiovascular nurse recorded all audio content using standard Mandarin, a consistent speaking speed, and plain language to minimize variability in oral explanation.

The printed materials were then paired with dedicated point codes. Codes were attached to the corresponding knowledge points in the self-developed manual, poster, and pill box. Each code was matched with the corresponding audio file and imported into the tap-and-talk pen system. When the pen recognized a code through infrared sensing, it automatically triggered the pre-recorded audio explanation for that point, enabling interactive but standardized self-learning. Because the pen was a commercial hardware carrier, no psychometric reliability or validity testing was conducted for the device itself. Before formal implementation, a one-week pilot test involving 20 post-PCI older adults was conducted to evaluate operational feasibility, clarity of audio content, ease of use, and user feedback. Minor wording and operation guidance were revised according to pilot feedback before the tool was finalized for clinical use.

**Fig. 2:**
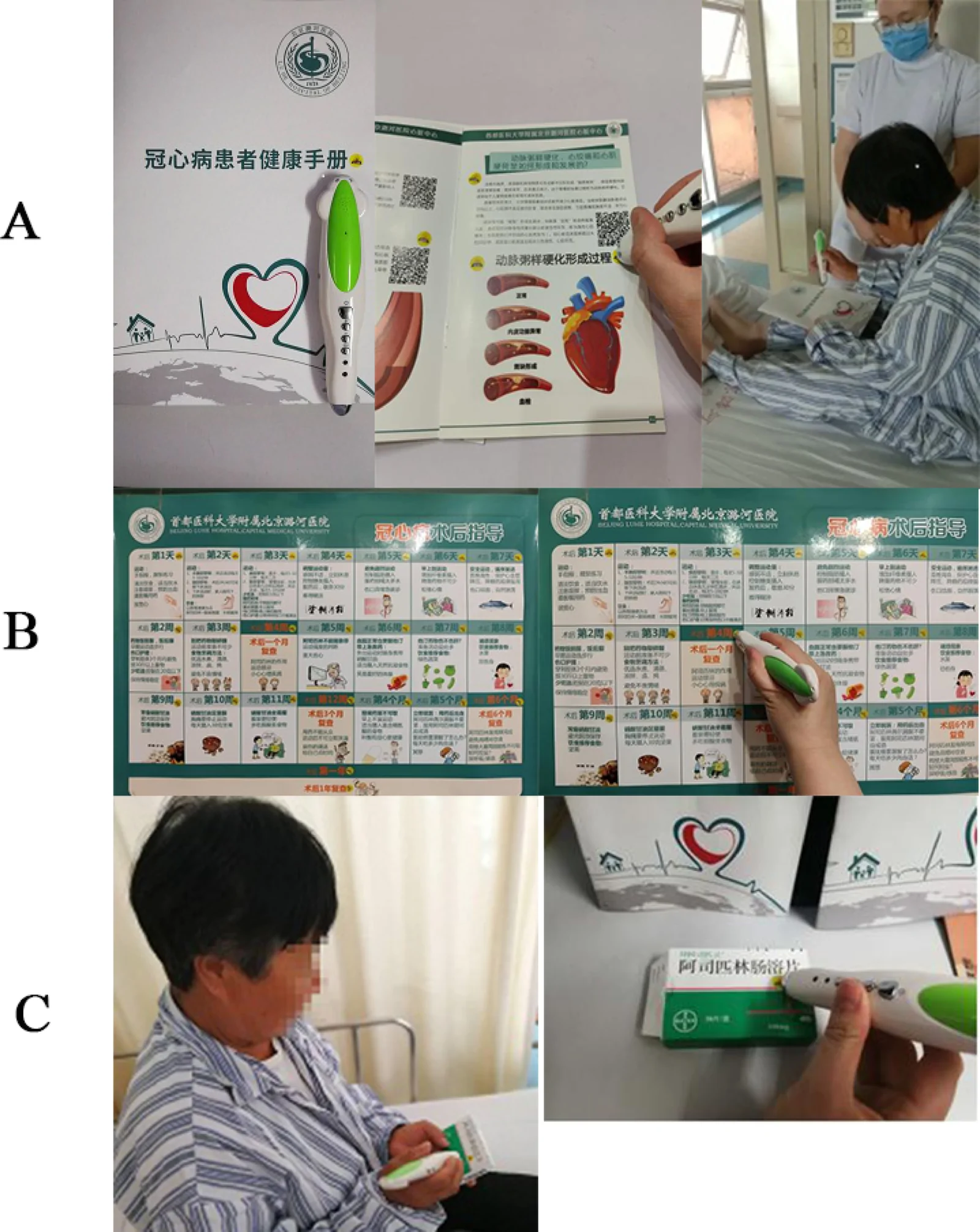
Handheld audio-guided patient education intervention for coronary heart disease. (**A**) Left: Cover of the Coronary Heart Disease Patient Handbook, featuring a heart and electrocardiogram design. Middle: Inner page showing the Atherosclerosis Formation Process with sequential diagrams of arterial plaque development (normal artery, fatty streak, fibrous plaque, calcified plaque, ruptured plaque). Right: A patient using the handheld reading pen to access audio explanations of the handbook content. (**B**) The Postoperative Guidance for Coronary Heart Disease chart, a structured timeline of recovery milestones from postoperative Day 1 to 12 months postoperatively, covering key aspects including diet, physical activity, medication adherence, and outpatient follow-up. A researcher demonstrates the use of the reading pen to access audio instructions for each recovery phase. (**C**) Left: A patient holds the reading pen and medication packaging. Right: Close-up of the reading pen interacting with the label of Aspirin Enteric-Coated Tablets, providing audio reminders for dosage and timing to support medication adherence

The intervention materials included: A printed manual identical in content to that used in the control group, a poster, a pill box, a Tap-and-Talk code, audio instructions regarding medication use, health education, and precautionary reminders. During hospitalization, participants received the same instructional content as those in the control group. On the day of discharge, patients were provided with a Tap-and-Talk pen and the corresponding educational materials. A health education nurse demonstrated how to operate the voice-guided educational tool, answered questions raised by patients during practice, and provided repeated demonstrations to ensure proficient use. The training lasted 15 to 20 min. trained both patients and their families in the use of the tool (Fig. 2.). The health education nurse provided training on using the health education tool to patients for 15 to 20 min and evaluated their mastery of the tool until they could use it proficiently.

The educational package was taken home for continued use. Weekly text message reminders were issued to encourage continued engagement. The total period of the intervention extended from admission to six months post-discharge. The tap-and-talk pen itself provided one-way delivery of pre-recorded educational information and did not support real-time questioning. To provide a channel for delayed two-way communication, research nurses sent one weekly text message after discharge to encourage engagement, review key knowledge points, and remind patients of the following week’s health tasks. Each message informed participants that if they had questions, discomfort, or difficulty understanding the educational content, they could reply directly to the message or call the department consultation telephone number. Research nurses collected all text-message replies and telephone inquiries, documented them, and responded individually within 24 h on working days.

#### Baseline survey

Questionnaires were distributed by the designated nurse. The baseline assessment consisted of three components: (1) a demographic and clinical data questionnaire, administered on the first day of admission and reviewed immediately for completeness; (2) a questionnaire assessing knowledge of disease rehabilitation in patients undergoing PCI and (3) a self-management behavior scale for CHD. The basic demographic and clinical data questionnaire was distributed on the first day of admission, collected on the spot by the responsible nurse after patients completed it, and checked for missing items. On the day before discharge, the responsible nurse assessed the patients’ knowledge of disease rehabilitation after percutaneous coronary intervention (PCI) and administered the coronary heart disease self-management behavior scale.

The instruments used are described below:

1. Basic data questionnaire

A self-developed questionnaire was used to collect demographic data and background information (Supplementary materials). The final version was developed through consultation with two cardiology specialists and one nursing specialist. The questionnaire included items on name, sex, telephone number, age, occupation, educational attainment, marital status, annual household income, medical insurance status, disease duration, medical history, and prior surgical knowledge.

2) Questionnaire on knowledge of disease rehabilitation in patients undergoing PCI

This self-developed questionnaire was designed based on a comprehensive literature review and clinical experience, with further refinement following review and revision by two cardiology experts with associated senior titles or above and three nursing experts. The final questionnaire comprised 15 items across three domains: content of post-PCI health education provided by healthcare personnel, patient knowledge of daily health maintenance, and awareness of regular follow-up practices. Each item was scored as 1 for a correct response and 0 for an incorrect response. Content validity, assessed by five experts (intermediate or higher titles with ≥ 10 years of cardiovascular experience), yielded an index of 0.91. A pilot study involving 20 post-PCI patients demonstrated good internal consistency, with a Cronbach’s α coefficient of 0.866.

3) CHD self-management behavior scale

Developed by Ren et al., this scale consists of 27 items. Items 1 to 26 are scored using a 5-point Likert scale (1 to 5). Scoring for item 2 [14]7 is based on vaccination behavior, with influenza vaccination scored as: 0 times = 0 points, 1–2 times = 1 point, 3–4 times = 2 points, 5–6 times = 3 points; and pneumonia vaccination scored as: 0 times = 0 points, 1–2 times = 2 points. The total score for item 27 is derived by summing the influenza and pneumonia vaccination scores. Higher total scores indicate better self-management behaviors. In the current study, the scale demonstrated a Cronbach’s α coefficient of 0.800.

#### Effectiveness evaluation

During the patient’s hospitalization, data collection included a general information questionnaire, a Disease Rehabilitation Knowledge Awareness Questionnaire for PCI Patients, and the Coronary Heart Disease (CHD) Self-Management Behavior Scale. Follow-up assessments were conducted at 1 month, 3 months, and 6 months after discharge. The follow-up content included the Disease Rehabilitation Knowledge Awareness Questionnaire for PCI Patients and the CHD Self-Management Behavior Scale. Additionally, a Health Education Tool Evaluation Form was completed by patients during the 6-month post-discharge follow-up. The incidence of cardiovascular and cerebrovascular events, as well as patient feedback regarding the health educational tools, were also documented. All follow-up assessments were conducted by two trained nurses who were blinded to group allocation. Health education nurses and patients were not blinded. Standardized instructions and explanations were provided to minimize variability in questionnaire interpretation. Data collection was carried out through telephone interviews and hospital visits.

Major Adverse Cardiovascular Events (MACE): The incidence of cardiovascular and cerebrovascular events within six months after discharge was documented using a standardized cardiovascular event record form. Events recorded included nonfatal myocardial infarction, repeat revascularization, cardiac death, and cerebrovascular disease.

Patient Evaluation of Health Educational Tools: A self-developed evaluation form was used to assess patients’ perceptions of the educational tools. The scale was developed following an extensive literature review and integrated specific features of the tools applied in this study. The item pool underwent iterative revisions through consultation with three clinical experts and one nursing research specialist. The final version consisted of 20 items across five dimensions: usefulness, accessibility, applicability, interest, and satisfaction. Each item was rated on a five-point Likert scale: strongly agree (5 points), agree (4 points), uncertain (3 points), disagree (2 points), and strongly disagree (1 point). Content validity was assessed by five experts with intermediate or higher professional titles and at least 10 years of cardiovascular clinical experience, yielding a content validity index of 0.880. A pilot test involving 20 patients undergoing PCI demonstrated acceptable internal consistency, with a Cronbach’s α coefficient of 0.788.

#### Statistical analysis methods

Statistical analyses was conducted using SPSS version 22.0. Normality of continuous data was assessed prior to analysis. Variables with a normal distribution are presented as mean ± standard deviation, whereas non-normally distributed variables were expressed as median values. Categorical data are presented as frequency and composition ratio. Between-group comparisons at identical time points were conducted using the chi-squared test and independent-sample *t*-test, while within-group comparisons at two time points were performed using the chi-square test and paired *t*-test. To analyze temporal trends across multiple time points between groups, the covariance matrix sphericity was tested. if the assumption of sphericity was met, univariate repeated-measures analysis of variance (ANOVA) was applied. If the sphericity assumption was violated, repeated-measures ANOVA with appropriate correction was used. A *p* value of < 0.05 was considered statistically significant.

### Ethical principles

This study was approved by the Ethics Committee of Beijing Luhe Hospital, Capital Medical University (Approval No.: 2020-LHKY-049-03). Written informed consent was obtained from all participants after detailed explanations were provided regarding the study objectives and relevance. Participation was voluntary, and participants retained the right to withdraw at any stage without impacting their ongoing medical care. To ensure patient confidentiality, hospitalization numbers were used in place of names. All research data and related data were stored securely and used solely for the purposes of this investigation. Upon study completion, participants in the control group were provided with an explanation of the intervention procedures applied in the intervention group. Relevant support was provided to these participants upon request.

### Quality control methods

Control of data bias: Standardized instructions and uniform explanations were applied during data collection to reduce variability in participant comprehension, promote earnest participation, and minimize data-related bias. The questionnaire design featured clearly defined content and measurement indicators.

Control of misclassification bias: Random allocation was performed at the ward level, assigning entire wards to either the intervention group or the control group.

Control of selection bias: Recruitment was conducted strictly in accordance with predefined inclusion and exclusion criteria to ensure representative sampling.

Control of response bias: The study objectives and relevance of the study were communicated clearly, and research procedures were designed to be simple and feasible. These measures were intended to enhance participant cooperation, reduce non-response and dropout rates, increase the effective sample size, and minimize loss to follow-up.

Control of contamination bias: Patients in the intervention and control groups were accommodated in separate wards. On the day of discharge, instruction in the use of the tap-and-talk health educational tool was provided exclusively to patients in the intervention group to prevent cross-group contamination.

## Results

### Patient enrollment and data comparison

A total of 50 patients were initially enrolled in both the intervention and control groups. During follow-up, one patient in the intervention group experienced a sudden cerebral infarction three months post-discharge and withdrew from the study. In the control group, one patient sought treatment at another hospital and declined follow-up one month post-discharge, resulting in loss to follow-up, while two additional patients discontinued participation due to sudden cerebral infarction occurring one and three months after discharge, respectively. The overall dropout rate was 4%. Ultimately, 49 patients in the intervention group and 47 patients in the control group completed the study.

The baseline characteristics of the two groups before intervention were compared as follows: the mean age of participants was 71.23 ± 4.923 years in the experimental group and 70.26 ± 4.046 years in the control group. The age distribution was balanced between the two groups, with no statistically significant difference (t = 1.605, *P* = 0.112). In terms of gender composition, the experimental group consisted of 24 males (48.98%) and 25 females (51.02%), while the control group included 29 males (61.70%) and 18 females (38.30%). There was no significant difference in gender distribution between the two groups (χ 2 = 1.570, *P* = 0.210).

No statistically significant differences were identified between participants who completed the study and those who withdrew, in terms of demographic, sociological, or disease-related parameters (*p* > 0.05) (Table 1). Additionally, no statistically significant differences were observed between the intervention and control groups at baseline for these same parameters (*p* > 0.05) (Table 2).

**Table 1:** Comparison of demographic and disease-related characteristics between patients initially enrolled and those who completed the study

Item		Patients initially enrolled (*n* = 100)	Patients who completed the study (*n* = 96)	t/χ^2^	*P*		
Age		71.23 ± 4.923	70.26 ± 4.046	1.605^a^	0.112		
Sex	Male	56	53	0.012^b^	0.911		
	Female	44	43				
Marital status	Divorced	1	1	0. 298^c^	1.000		
	Married	94	90				
	Widowed	5	5				
Educational attainment	Primary school and below	37	36	0.082^c^	1.000		
	Junior high school/high school	59	56				
	College and above	4	4				
Living conditions	Living alone	7	7	0.021^b^	0.990		
	Living with spouse	80	76				
	Living with children	13	13				
Payment methods	Urban employee basic medical insurance	43	39	0.114^b^	0.990		
	Urban resident basic medical insurance	42	42				
	New rural cooperative medical insurance	10	10				
	Out-of-pocket	5	5				
Coronary artery disease	Undiagnosed	62	61	0.189^c^		0.955	
	Diagnosed within 1 year	2	2				
	Diagnosed for more than 1 year	36	33				
Vascular stent therapy	Yes	40	39	0.008^b^		0.929	
	No	60	57				
Coronary artery bypass graft therapy	Yes	4	4	0.003^c^		0.953	
	No	96	92				
Arrhythmia	Yes	13	13	0.012^b^		0.911	
	No	87	83				
Hypertension	Yes	82	78	0.018^b^		0.892	
	No	18	18				
Hyperlipidemia	Yes	52	48	0.078^b^		0.779	
	No	48	48				
Diabetes	Yes	39	37	0.004^b^		0.948	
	No	61	59				
Cerebrovascular disease	Yes	24	24	0.026^b^		0.871	
	No	76	72				
Smoking	Yes	24	21	0.125^b^		0.724	
	No	76	75				

a indicates an independent sample t-test; b indicates a chi-squared test; c indicates Fisher’s exact test

**Table 2:** Comparison of demographic and disease-related characteristics between the intervention and control groups

Item		Intervention group (*n* = 49)	Control group (*n* = 47)	t/χ^2^	*P*
Age		71.23 ± 4.923	70.26 ± 4.046	1.605^a^	0.112
Sex	Male	24	29	1.570^b^	0.210
	Female	25	18		
Marital status	Divorced	0	1	2.546^c^	0.362
	Married	45	45		
	Widowed	4	1		
Educational attainment	Primary school and below	20	16	1.689^c^	0.445
	Junior high school/high school	26	30		
	College and above	3	1		
Living conditions	Living alone	4	3	0.267^c^	1.000
	Living with spouse	38	38		
	Living with children	7	6		
Payment methods	Urban employee basic medical insurance	18	21	1.724^c^	0.688
	Urban resident basic medical insurance	24	18		
	New rural cooperative medical insurance	4	6		
	Out-of-pocket	3	2		
Coronary artery disease	Undiagnosed	35	26	3.654^c^	0.123
	Diagnosed within 1 year	0	2		
	Diagnosed for more than 1 year	14	19		
Vascular stent therapy	Yes	15	24	4.160^b^	0.061
	No	34	23		
Coronary artery bypass graft therapy	Yes	3	1		0.617
	No	46	46		
Arrhythmia	Yes	6	7	0.144^b^	0.771
	No	43	40		
Hypertension	Yes	38	40	0.899^b^	0.436
	No	11	7		
Hyperlipidemia	Yes	22	26	1.042^b^	0.307
	No	27	21		
Diabetes	Yes	19	18	0.002^b^	1.000
	No	30	29		
Cerebrovascular disease	Yes	11	13	0.347^b^	0.640
	No	38	34		
Smoking	Yes	7	14	3.373^b^	0.085
	No	42	33		

a indicates an independent sample t-test; b indicates a chi-squared test; c indicates Fisher’s exact test

### Comparison of CHD knowledge levels between groups

**Fig. 3:**
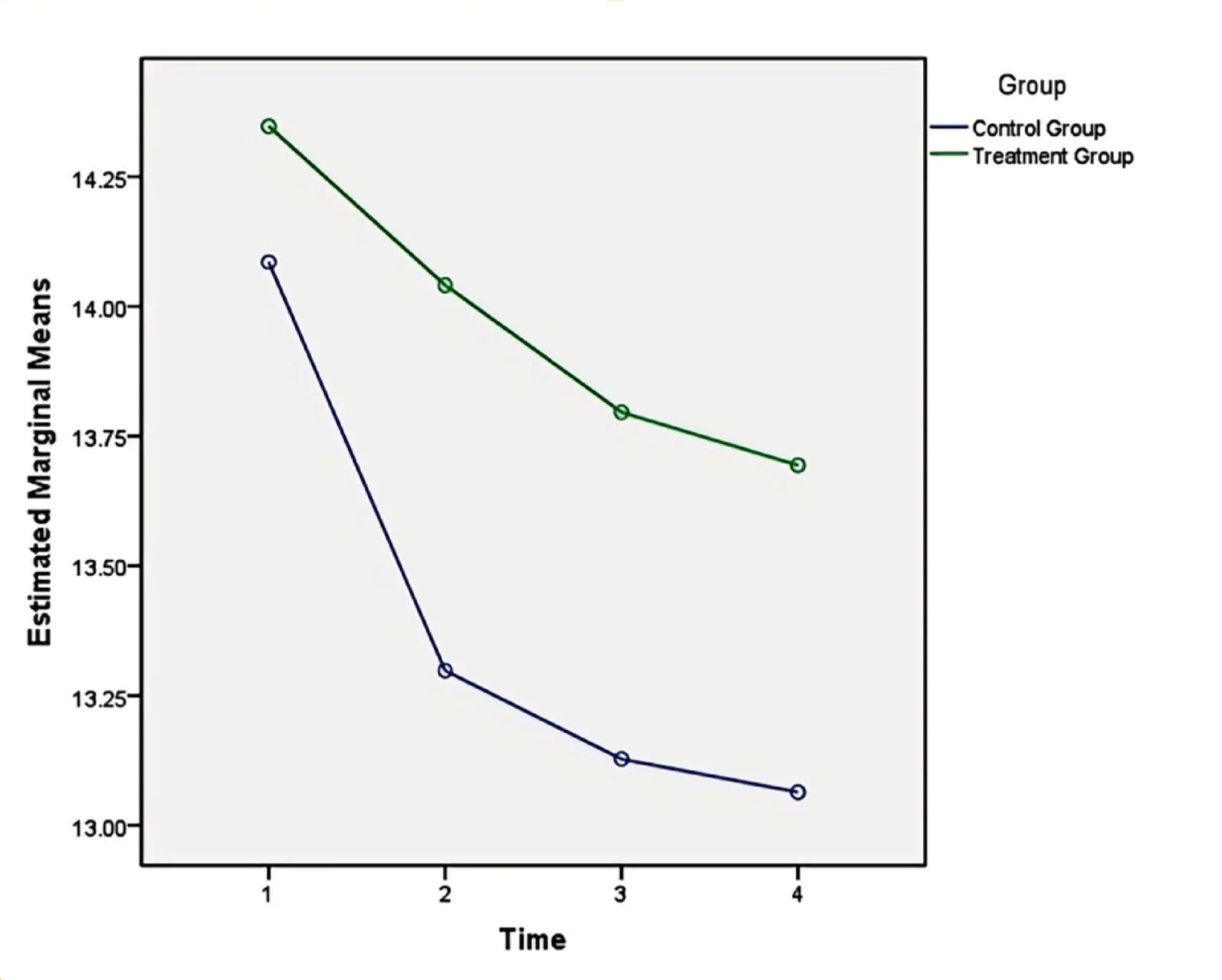
Changes in coronary heart disease–related knowledge scores between the intervention and control groups at baseline, 1 month, 3 months, and 6 months after discharge. Time on the horizontal axis: 1 = baseline; 2 = 1 month; 3 = 3 months; 4 = 6 months

A significant group × time interaction effect was detected for CHD-related knowledge (FTime×Group = 9.026, *p* < 0.001), with main effects of group (FGroup = 11.607, *p* = 0.001) and time (FTime = 124.067, *p* < 0.001). At baseline, knowledge scores were similar between groups (intervention: 14.34 ± 0.73; control: 14.09 ± 0.75, *p* > 0.05). However, at 1 month, 3 months, and 6 months post-intervention, the intervention group achieved significantly higher scores than the control group: 14.02 ± 0.82 vs. 13.30 ± 0.83 (1 month), 13.79 ± 0.75 vs. 13.13 ± 0.90 (3 months), and 13.70 ± 0.66 vs. 13.06 ± 0.89 (6 months) (all *p* < 0.01). Both groups showed a mild downward trend in knowledge scores over time, but the rate of decline was significantly slower in the intervention group (Table 3; Fig. 3).

**Table 3:** Comparison of health education outcomes between the intervention and control groups

	Baseline	One month	Three months	Six months	F_Group_ /*P*	F_Time_ /*P*	F_Time×Group_/*P*
1. Coronary heart disease–related knowledge score					11.607/0.001	124.067/<0.001	9.026/<0.001
Control group (*n* = 47)	14.09 ± 0.75	13.30 ± 0.83*	13.13 ± 0.90*	13.06 ± 0.89*			
Intervention group (*n* = 49)	14.34 ± 0.73	14.02 ± 0.82	13.79 ± 0.75	13.70 ± 0.66			
2. Self-management behavior score					195.188/<0.001	124.067/<0.001	9.026/<0.001
Control group (*n* = 47)	70.98 ± 6.43	82.06 ± 6.65*	81.21 ± 6.62*	81.06 ± 6.73*			
Intervention group (*n* = 49)	72.70 ± 8.49	89.06 ± 10.64	89.21 ± 7.51	89.15 ± 7.62			
Bad habit management behavior score					1.234/0.272	73.976/<0.001	8.400/0.001
Control group (*n* = 47)	12.57 ± 2.76	13.32 ± 2.72	13.53 ± 2.50	13.60 ± 2.44			
Intervention group (*n* = 49)	12.47 ± 2.55	13.89 ± 2.78	14.47 ± 1.90	14.55 ± 1.93			
Life management behavior score					2.878/0.097	130.959/<0.001	6.233/0.006
Control group (*n* = 47)	13.72 ± 2.13	15.06 ± 1.92	15.04 ± 1.84*	15.00 ± 1.83*			
Intervention group (*n* = 49)	13.79 ± 1.71	15.62 ± 1.70	15.91 ± 1.38	16.02 ± 1.38			
Symptom management behavior score					2.149/0.045	395.049/<0.001	4.525/0.02
Control group (*n* = 47)	8.09 ± 0.78	11.09 ± 1.37	11.04 ± 1.36	10.98 ± 1.48			
Intervention group (*n* = 49)	7.94 ± 1.21	11.74 ± 2.20	11.62 ± 1.96	11.55 ± 1.89			
Disease knowledge management behavior score					12.701/0.001	250.943/<0.001	25.223/<0.001
Control group (*n* = 47)	9.49 ± 1.69	11.36 ± 1.51*	11.34 ± 1.54*	11.26 ± 1.58*			
Intervention group (*n* = 49)	9.57 ± 1.74	13.04 ± 2.46	13.11 ± 1.88	13.00 ± 1.98			
Treatment adherence management behavior score					7.560/0.009	321.108/<0.001	1.802/0.168
Control group (*n* = 47)	7.57 ± 0.90	10.06 ± 0.90*	9.19 ± 0.83*	9.15 ± 0.88*			
Intervention group (*n* = 49)	7.77 ± 1.13	10.57 ± 1.10	9.74 ± 0.71	9.62 ± 0.77			
Emergency management behavior score					0.922/0.342	182.942/<0.001	14.965/<0.001
Control group (*n* = 47)	6.79 ± 1.10*	8.57 ± 1.15	8.51 ± 1.16*	8.55 ± 1.12*			
Intervention group (*n* = 49)	6.04 ± 1.41	9.02 ± 1.36	9.11 ± 1.09	9.06 ± 1.13			
Emotional and cognitive behavior score					74.677/<0.001	0.131/0.89	2.217/0.114
Control group (*n* = 47)	12.74 ± 1.33*	12.60 ± 1.26*	12.55 ± 1.34*	12.53 ± 1.35*			
Intervention group (*n* = 49)	15.13 ± 1.91	15.17 ± 2.16	15.26 ± 1.88	15.32 ± 1.84			

*Compared with the intervention group, *p* < 0.05

### Comparison of self-management behaviors between groups

**Fig. 4:**
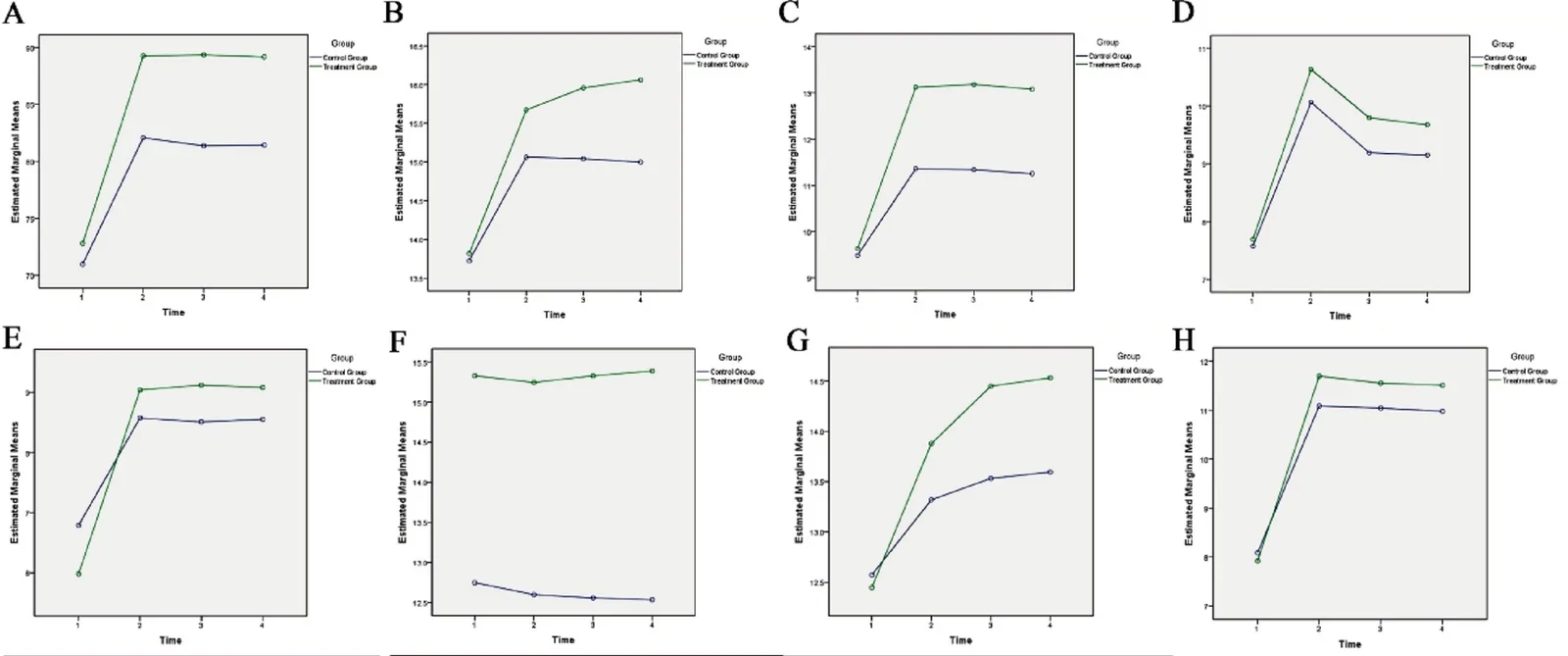
Changes in total and dimension-specific scores of the self-management behavior scale between the two groups at different time points: **A**. Total self-management behavior score. **B**. Lifestyle management behavior score. **C**. Disease knowledge management behavior score. **D**. Treatment adherence management behavior score. **E**. Emergency management behavior score. **F.** Emotional and cognitive management behavior score. **G**. Bad habit management behavior score. **H**. Symptom management behavior score. Time on the horizontal axis: 1 = baseline; 2 = 1 month; 3 = 3 months; 4 = 6 months

For total self-management behavior scores, a significant group × time interaction was observed (FTime×Group = 9.026, *p* < 0.001), along with main effects of group (FGroup = 195.188, *p* < 0.001) and time (FTime = 124.067, *p* < 0.001). The intervention group consistently achieved higher total scores than the control group at all follow-up time points: 89.06 ± 10.64 vs. 82.06 ± 6.65 (1 month), 89.21 ± 7.51 vs. 81.21 ± 6.62 (3 months), and 89.15 ± 7.62 vs. 81.06 ± 6.73 (6 months) (all *p* < 0.01) (Table 3; Fig. 4A).

Dimension-specific findings were as follows:

Lifestyle management behavior: A significant group × time interaction was identified (FTime×Group = 6.233, *p* = 0.006), with main effects of time (FTime = 130.959, *p* < 0.001) but not group (FGroup = 2.878, *p* = 0.097). The intervention group had significantly higher scores at 3 months (15.91 ± 1.38 vs. 15.04 ± 1.84) and 6 months (16.02 ± 1.38 vs. 15.00 ± 1.83) compared with the control group (both *p* < 0.01), while the control group’s scores plateaued after 1 month (Table 3; Fig. 4B).

Disease knowledge management behavior: A significant group × time interaction was observed (FTime×Group = 25.223, *p* < 0.001), with main effects of group (FGroup = 12.701, *p* = 0.001) and time (FTime = 250.943, *p* < 0.001). The intervention group scored significantly higher than the control group at all follow-up time points: 13.04 ± 2.46 vs. 11.36 ± 1.51 (1 month), 13.11 ± 1.88 vs. 11.34 ± 1.54 (3 months), and 13.00 ± 1.98 vs. 11.26 ± 1.58 (6 months) (all *p* < 0.01) (Table 3; Fig. 4C).

Treatment adherence management behavior: Main effects of group (FGroup = 7.560, *p* = 0.009) and time (FTime = 321.108, *p* < 0.001) were detected, but the group × time interaction was not significant (FTime×Group = 1.802, *p* = 0.168). The intervention group showed significantly higher scores than the control group at 1 month (10.57 ± 1.10 vs. 10.06 ± 0.90), 3 months (9.74 ± 0.71 vs. 9.19 ± 0.83), and 6 months (9.62 ± 0.77 vs. 9.15 ± 0.88) (all *p* < 0.05) (Table 3; Fig. 4D).

Emergency management behavior: A significant group × time interaction was found (FTime×Group = 14.965, *p* < 0.001), with main effects of time (FTime = 182.942, *p* < 0.001) but not group (FGroup = 0.922, *p* = 0.342). Although the intervention group had lower baseline scores (6.04 ± 1.41 vs. 6.79 ± 1.10, *p* < 0.05), their scores surpassed the control group at 3 months (9.11 ± 1.09 vs. 8.51 ± 1.16) and 6 months (9.06 ± 1.13 vs. 8.55 ± 1.12) (both *p* < 0.05) (Table 3; Fig. 4E).

Emotional and cognitive behavior: A significant main effect of group was observed (FGroup = 74.677, *p* < 0.001), but main effects of time (FTime = 0.131, *p* = 0.890) and group × time interaction (FTime×Group = 2.217, *p* = 0.114) were not significant. The intervention group scored significantly higher than the control group at all time points (baseline: 15.13 ± 1.91 vs. 12.74 ± 1.33; 1 month: 15.17 ± 2.16 vs. 12.60 ± 1.26; 3 months: 15.26 ± 1.88 vs. 12.55 ± 1.34; 6 months: 15.32 ± 1.84 vs. 12.53 ± 1.35; all *p* < 0.05) (Table 3; Fig. 4F).

Bad habit management behavior: A significant group × time interaction was detected (FTime×Group = 8.400, *p* = 0.001), with a main effect of time (FTime = 73.976, *p* < 0.001) but not group (FGroup = 1.234, *p* = 0.272). The intervention group had numerically higher scores than the control group at all follow-up time points (13.89 ± 2.78 vs. 13.32 ± 2.72 at 1 month; 14.47 ± 1.90 vs. 13.53 ± 2.50 at 3 months; 14.55 ± 1.93 vs. 13.60 ± 2.44 at 6 months), but the differences did not reach statistical significance (all *p* > 0.05) (Table 3; Fig. 4G).

Symptom management behavior: A significant main effect of time was found (FTime = 395.049, *p* < 0.001), main effects of group (FGroup = 2.149, *p* = 0.045) and group × time interaction (FTime×Group = 4.525, *p* = 0.020), they were statistically significant. No significant between-group differences were observed at any time point (all *p* > 0.05) (Table 3; Fig. 4H).

### Adverse cardiovascular and cerebrovascular events during the intervention period in both groups

Within 6 months post-discharge, 3 adverse cardiovascular events (all cerebral infarctions) were documented across both groups. One event occurred in the intervention group (incidence: 2.04%), and two events occurred in the control group (incidence: 4.26%). No statistically significant difference in event rates was observed between groups (χ²=0.347, *p* = 0.640).

### Patient acceptance of the tap-and-talk health educational tool

A total of 49 valid evaluation questionnaires were collected from the intervention group. The tool received high acceptance ratings, with an overall mean score of 96.04 ± 2.61 (range: 20–100). Dimension-specific mean scores (± SD) were as follows: accessibility (4.97 ± 0.08), satisfaction (4.96 ± 0.14), usefulness (4.95 ± 0.13), interest (4.61 ± 0.31), and applicability (4.48 ± 0.28) (all scored on a 5-point Likert scale). Participants reported high ease of use, willingness to engage, and intention for continued use of the tool (Table 4).

**Table 4:** Patient acceptance of the tap-and-talk health educational tool across five evaluation dimensions

Dimension	Score range	Total dimension score ($\overline{x}$± s)	Mean score of items ($\overline{x}$± s)
Usefulness	4–20	19.82 ± 0.53	4.95 ± 0.13
Accessibility	4–20	19.88 ± 0.33	4.97 ± 0.08
Applicability	3–15	13.43 ± 0.84	4.48 ± 0.28
Interest	5–25	23.06 ± 1.56	4.61 ± 0.31
Satisfaction	4–20	19.86 ± 0.54	4.96 ± 0.14
Overall score	20–100	96.04 ± 2.61	4.80 ± 0.49

## Discussion

### Effect of the tap-and-talk tool on cardiovascular knowledge retention in older adults post-PCI

Findings from this study demonstrated that disease-related knowledge among older adult patients was influenced by both group assignment and time, with a statistically significant interaction effect. The two health education strategies produced distinct effects in terms of CHD knowledge levels. At baseline, following standardized inpatient education, no differences in knowledge scores were observed between groups. However, at 1, 3, and 6 months after discharge, patients in the intervention group consistently achieved higher scores than those in the control group. While a minor decline in knowledge was observed in the intervention group over time, the control group exhibited a more pronounced decrease.

Inpatient education serves as the primary channel through which patients with CHD acquire relevant knowledge [11]. The hospital setting allows for systematic, comprehensive, and concentrated health education delivered by trained medical staff. In this study, both groups received individualized, nurse-led oral instruction during hospitalization, accounting for similar baseline knowledge scores. The observed decline in knowledge after discharge aligns with the established principles of memory decay, as described by the Ebbinghaus forgetting curve. According to this model, memory loss occurs rapidly in the initial phase and slows over time, reflecting the principle of “fast at first, slow thereafter” [15]. This effect is particularly pronounced in older adults, whose memory retention is generally more limited. Previous research has reported that only approximately 20% of content is retained after 31 days. [16–17] Thus, the slower rate of decline observed in the intervention group suggests that the tap-and-talk tool provided repeated reinforcement of health information, which may have facilitated better long-term retention.

Compared with conventional health education, the tap-and-talk tool improved understanding of disease for the patients and contributed to sustained cognitive retention. Its user-friendly design enabled repeated practice, allowing patients to revisit educational content as needed. This iterative exposure reinforced coronary heart disease (CHD) knowledge and supported long-term retention. By facilitating active engagement with the educational material, the tool helped counteract memory decline and reinforced comprehension. In older adults with CHD, improved cognition supports healthier behaviors, reduces hospitalization rates, and improves prognosis [18]. In this study, individualized guidance was provided to patients and their families during hospitalization, with ongoing encouragement to incorporate the tool into daily routines. Post-discharge follow-up conducted by nursing staff served to reinforce the principles of active learning and helped sustain disease-related knowledge over time. Proactive communication during follow-up promoted ongoing use of the tap-and-talk tool, facilitated consistent engagement with educational content, mitigated memory decline, and enhanced disease-related cognition.

Internationally, studies on digital health education for post-PCI patients have highlighted the value of targeted interventions for knowledge retention. Chai et al. [10] evaluated conventional text-based patient education in cardiac rehabilitation and reported a 10% decline in disease knowledge within 6 months, which is more pronounced than the mild 4.5% decline observed in our intervention group. This difference underscores the advantage of our voice-guided tool’s repeated reinforcement design, which aligns with the “teach-back” principle validated by Peter et al. [19] to improve information retention in patients hospitalized with heart failure. A meta-analysis and systematic review showed that empowerment education focusing on full and comprehensive rehabilitation theory education could increase the knowledge of coronary heart disease in patients after PCI [20]. Unlike Chinese studies focusing on younger post-PCI cohorts (mean age 59 years) [10], our study targets a more elderly population (mean age 70–71 years), demonstrating that voice-guided interactive tools can effectively mitigate age-related memory decay [7, 9].

Locally, domestic research has consistently highlighted the limitations of traditional health education in sustaining disease-related knowledge among post-PCI patients [11, 14]. Specifically, Yin et al. [11] documented that only 30% of patients retained key health knowledge six months after discharge, a finding that stands in stark contrast to our intervention group, which maintained 95% of its baseline knowledge scores over the same period. This notable discrepancy stems primarily from the superior accessibility of our tap-and-talk tool: unlike nurse-led follow-up— which is often constrained by time and geographical barriers [21] our tool enables patients to independently revisit audio-based educational content at their convenience. This feature directly addresses the low participation rate in hospital-based cardiac rehabilitation programs across China [15], a critical gap that traditional education fails to mitigate. Furthermore, most domestic studies lack age-specific design considerations [14], overlooking the unique cognitive characteristics of older adults, such as age-related memory decline and limited digital literacy [9]. In contrast, our tool’s simplified tap operation and voice narration are specifically tailored to these needs, ensuring that the target population can easily engage with and benefit from the educational content.

### Effect of the tap-and-talk tool on self-management behaviors in older adults post-PCI

This study demonstrated that self-management behaviors were influenced by both time and group assignment, with a significant interaction effect. The two health education strategies demonstrated different impacts on self-management outcomes. Analysis across the seven dimensions of the self-management behavior scale indicated that, following intervention, participants in the intervention group achieved significantly higher scores than those in the control group in the areas of life management, disease knowledge management, treatment adherence, and emergency management, with all differences reaching statistical significance. Post-PCI health knowledge directly influences patient behavior. Sufficient understanding of disease-related information and awareness of its significance support appropriate behavioral responses, thereby facilitating recovery. Over time, improvements in self-management ability were observed in both groups, consistent with the positive effects of continued exposure to health education delivered by healthcare professionals. Such interventions promote healthier lifestyle patterns and contribute to improved quality of life in individuals with CHD [21]. 

The tap-and-talk health educational tool demonstrated greater effectiveness than conventional education methods. In the intervention group, life management scores continued to increase throughout the intervention period, whereas in the control group, scores peaked at one month and subsequently declined. This divergence was associated with the broader scope of the tap-and-talk tool, which emphasized self-management across lifestyle modification, disease-specific care, treatment monitoring, and emergency response, while its repeated voice-based delivery reinforced comprehension and retention of educational content. Previous research has shown that auditory repetition can activate semantic memory in older adults to levels comparable with younger individuals following repeated exposure [19]. Repeated practice using the tap-and-talk tool likely contributed to enhance self-regulation in daily health behaviors. However, improvements in other self-management dimensions were less pronounced. Scores in several areas plateaued or declined slightly at three and six months post-intervention. While follow-up communication and encouragement were provided by nurses to maintain engagement with the tap-and-talk tool, the limited frequency of post-discharge follow-up—restricted to three scheduled sessions—may not have been sufficient to sustain learning motivation or provide timely feedback on patient progress.

Regarding bad habit management and symptom management, scores in the intervention group remained higher than those in the control group at one, three, and six months after intervention, although the differences did not reach statistical significance. Similarly, no significant differences were observed in emotional and cognitive management behaviors between the two groups. Although the educational program emphasized the importance of eliminating unhealthy habits, behavioral change remained limited. Many patients with CHD, despite awareness of associated risks, demonstrated sufficient intrinsic motivation to modify established behaviors. The inability of the tap-and-talk tool to influence intrinsic motivation represents a key limitation. Furthermore, the tool did not provide individualized strategies for symptom management or emotional and cognitive support, limiting the potential for personalization in these dimensions within the existing framework of tap-and-talk learning. To address this situation, it is necessary to strengthen patients’ intrinsic motivation, provide individualized support for symptom and emotion management, and sustain intervention effects through personalized incentives, tool optimization, improved follow-up, and multidisciplinary collaboration.

Internationally, digital interventions for post-PCI self-management have shown varying effectiveness. A U.S.-based randomized controlled trial demonstrated that employing the “teach-back” method for patient education significantly reduced the 30-day hospital readmission rate in heart failure patients (White et al., 2013) [22]. In contrast, the intervention in our study, utilizing the tap-and-talk tool, achieved a 21.2% improvement in the adherence management behavior score over a 6-month period. This enhanced effect may stem from our tool’s combination of weekly text reminders and on-demand audio content, which provides continuous reinforcement beyond in-person sessions. In China, Chai et al. [10] noted that conventional education failed to sustain self-management improvements beyond 3 months, whereas our intervention group maintained high scores at 6 months, reflecting the tool’s ability to support long-term behavioral change.

Locally, Zhu et al. [21] reported that continuous nursing interventions improved total self-management scores by 14% in post-PCI patients, compared to a 22.9% improvement in our intervention group. This difference is likely due to our tool’ s comprehensive coverage of self-management domains—including emergency response and disease knowledge management—that are often underaddressed in traditional nursing interventions [13]. Unlike mobile app-based interventions in China that require smartphone proficiency [11], our tap-and-talk tool is accessible to older adults with limited digital skills [9], resulting in higher sustained engagement (89% of participants used it ≥ 3 times weekly).

### Acceptability of the tap-and-talk health educational tool among older adults post-PCI

Older adults often experience age-related declines in cognitive function and memory, which can impair their ability to comprehend and engage with conventional, text-based health education. These cognitive changes may also reduce receptivity to novel educational formats. The tap-and-talk tool was designed to address these characteristics by providing a simple and accessible educational method. Findings from the patient feedback survey indicated high levels of acceptance of the tool among participants in the intervention group. Mean scores across all five assessed dimensions—usefulness, accessibility, applicability, interest, and satisfaction—exceeded 4.48 on a 5-point Likert scale. These results suggest that the format and delivery of the tap-and-talk tool were well-suited to the cognitive and perceptual characteristics of older adult patients.

### Positive and negative impacts of the intervention on post-PCI patients

Beyond the primary outcomes of improved knowledge and self-management, the intervention offers multiple additional benefits. First, the tool’s high acceptability (mean score 96.04 ± 2.61) reflects its user-centric design—89% of participants reported using it ≥ 3 times per week, indicating sustained engagement that complements traditional cardiac rehabilitation. This independent access reduces reliance on healthcare providers and family caregivers, enhancing patients’ sense of autonomy, which is particularly valuable for older adults transitioning from hospital to home care. Second, the intervention showed a trend toward reducing adverse cardiovascular events (2% in the intervention group vs. 4.08% in the control group) [3], aligning with previous evidence that improved self-management lowers MACE risk [4, 18]. Although the difference did not reach statistical significance, it suggests potential long-term clinical value worthy of larger-scale validation. Third, the tool’s standardized content ensures consistency in health education delivery, addressing the variability in quality observed in nurse-led conventional education [11], which is crucial for ensuring uniform secondary prevention standards across settings.

Several methodological limitations should be acknowledged. First, although cluster randomization by ward was implemented to reduce contamination bias, the analytical approach did not account for clustering effects (e.g., through mixed-effects models or generalized estimating equations), which may lead to underestimation of standard errors and overly optimistic p-values. Second, despite randomization, baseline imbalances were observed in certain self-management dimensions (e.g., emotional and cognitive management, emergency management), which may have influenced the interpretation of group effects; these imbalances were not statistically adjusted in the current analysis. Third, multiple comparisons across the seven self-management domains were conducted without correction for Type I error, increasing the risk of false-positive findings. Fourth, a self-developed knowledge questionnaire was used rather than a validated instrument such as the CADE-Q or ACS Knowledge Tool, which limits comparability with other studies and may introduce measurement bias. Fifth, the use of convenience sampling and the single-center design restrict the generalizability of the findings to broader populations and settings. Sixth, the development and implementation of the tap-and-talk tool required interdisciplinary collaboration; however, limitations in available resources constrained material selection and device scalability. Additionally, the audio-visual content requires continuous updates to remain consistent with evolving clinical guidelines, necessitating sustained maintenance and revision. Finally, the study included only older adult patients undergoing PCI within a single cardiology department. Future multi-center studies with cluster-adjusted analyses, validated outcome measures, a priori correction for multiple comparisons, and longer follow-up are needed to confirm the efficacy and generalizability of the tap-and-talk tool.

The results of this study demonstrated that the Voice-Guided Tap-and-Talk health education tool effectively enhanced the self-management capabilities of elderly patients following PCI. We propose that this tool holds considerable application potential in broader patient populations and clinical settings. Owing to its convenience and standardization, it is theoretically applicable to other populations requiring long-term chronic disease management (e.g., diabetes mellitus, heart failure) or postoperative rehabilitation. Particularly in regions with relatively inadequate primary medical resources, this tool is anticipated to serve as a low-cost and high-efficiency auxiliary approach for out-of-hospital management, thereby alleviating the workload of medical and nursing staff.

### Study limitations

The development and implementation of the tap-and-talk health educational tool necessitated an interdisciplinary approach involving collaboration among experts in medicine, electronic data technology, and network security. However, limitations in available resources constrained both material selection and device scalability. In addition, the audio-visual content requires continuous updates to remain consistent with evolving clinical guidelines, thereby necessitating sustained maintenance and revision. Furthermore, this study was limited by its single-center design and the inclusion of only older adult patients undergoing PCI within a single cardiology department. These factors limit the generalizability of the findings. Broader, multi-center investigations are required to comprehensively validate the effectiveness of the tap-and-talk tool in older adult patients undergoing PCI.

In addition, the tap-and-talk pen provided pre-recorded audio explanations and therefore functioned as a one-way educational tool during immediate use. Although weekly text-message reminders and telephone consultation provided a delayed two-way communication channel, this approach was not equivalent to real-time face-to-face communication or synchronous instant messaging. Delayed feedback may have reduced some patients’ willingness to ask questions promptly and may have limited the depth of individualized education, particularly for older adults with lower comprehension ability. This limitation may have led to underestimation of the intervention effect on knowledge acquisition and self-management behavior. Future studies should consider incorporating real-time bidirectional communication to optimize the educational effect.

## Conclusion

This randomized controlled trial evaluated the effectiveness of a tap-and-talk health educational tool, delivered in audiobook format with voice narration, in enhancing knowledge acquisition and self-management behaviors in older adults following PCI. The results demonstrated that use of the tool significantly improved both disease-related knowledge and self-management capacities at 1, 3, and 6 months post-discharge. Additionally, participants in the intervention group reported high levels of acceptance, indicating that the tool was well-suited to the educational needs of older adult patients with coronary heart disease. These findings support the integration of voice-guided, user-friendly educational tools as a complementary strategy in post-PCI patient education. Future research should verify the tool’s generalizability through multi-center studies and long-term follow-up beyond 6 months. Policy-wise, incorporating the tool into national cardiac rehabilitation guidelines, expanding medical insurance coverage, and standardizing content updates could facilitate its clinical implementation, addressing gaps in China’s cardiac rehabilitation services.

## Supplementary Information

Below is the link to the electronic supplementary material.

Supplementary Material 1

## Data Availability

All relevant data are within the paper.

## Abbreviations

CVD: Cardiovascular disease
PCI: Percutaneous coronary intervention
MACE: Major adverse cardiovascular events
CHD: Coronary heart disease
