# Supplementary material for: Effectiveness of a voice-guided tap-and-talk educational tool in enhancing cardiovascular knowledge and self-management in aged patients following percutaneous coronary intervention
Source: J Cardiothorac Surg. 2026 Jul 22;21:812. doi: 10.1186/s13019-026-04546-3 (PMC13678935; doi:10.1186/s13019-026-04546-3)
Supplement: Supplementary file 1 [file 13019_2026_4546_MOESM1_ESM.docx]

**Questionnaire of knowledge about disease rehabilitation in patients undergoing PCI**

**Part 1. Disease-related knowledge**

1. What is coronary heart disease? （ ）

A Coronary heart disease is a heart disease caused by myocardial ischemia, hypoxia or myocardial necrosis due to coronary artery stenosis or obstruction.

B Coronary heart disease is a heart disease caused by myocardial ischemia, hypoxia or myocardial necrosis due to aortic stenosis or obstruction.

C coronary heart disease is a kind of heart disease with myocardial ischemia and hypoxia or myocardial necrosis caused by pulmonary artery stenosis or obstruction.

2. The unchangeable risk factors of coronary heart disease are ()

A Smoking B Hypertension C Aging

3. Percutaneous coronary intervention (PCI) is ()

A doctor treats coronary artery stenosis or occlusion through catheters and other instruments through the arteries of upper or lower limbs.The blood vessel lumen is restored and the blood flow is unblocked again.

After thoracotomy, Doctor B treated the coronary artery stenosis or occlusion, so that the vascular lumen was restored and the blood flow was smooth again.

C doctors bypass coronary artery stenosis or occlusion through catheters and other instruments through the arteries of upper or lower limbs.The blood vessel lumen is restored and the blood flow is unblocked again.

4. Perioperative PCI considerations do not include ()

A. Fasting water before operation B. Medication according to medical supervision C. Cleaning the skin

5. What is the purpose of drinking 1500ml of water on the day after PCI? （ ）

A Prevent contrast-induced nephropathy B Prevent urethritis C Prevent infection

**Part 2. Knowledge related to postoperative rehabilitation**

1. The purpose of taking double antiplatelet drugs after PCI is ()

A Prevention of stent thrombosis B Prevention of bleeding C Prevention of infection

2. The name of the dual antiplatelet drug you are taking is ()

A Aspirin and clopidogrel bisulfate B Aspirin and ticagrelor C Aspirin and statins

3. The time requirement for taking double antiplatelet drugs is ()

A Two drugs for life B Clopidogrel for at least one year C Ticagrelor for at least one year

4. The effects of statins commonly used after PCI are ()

A Regulating blood sugar B Lowering blood pressure C Regulating blood lipid D Preventing blood coagulation

5. The secondary prevention of coronary heart disease does not include ()

A Excessive exercise B Quit smoking C Take medicine on time D Moderate exercise

6. The depth of chest compressions is ()

A 5-6 cm B 3-4 cm C 7-8 cm

7. The harm of quitting smoking does not include ()

A Increases lung disease B Risk factors for coronary heart disease C Causes atherosclerosis D Makes people live longer

**Part3. Relevant knowledge of postoperative reexamination**

8. The time of postoperative review is ()

A 2 months, 4 months, 8 months, 1 year, discomfort follow-up

B 1 month, 3 months, 6 months, 1 year, follow up for discomfort

C 3 months, 6 months, 9 months, 1 year, follow up for discomfort

9. The items of routine reexamination and laboratory test after discharge are ()

A. Further check the curative effect of the operation, adjust the dosage of drugs, and find new diseases as soon as possible.

B. Further treatment of diseases to avoid the discovery of new diseases.

C. Further treat the disease and avoid adjusting the dosage of the drug.

10. The emergency indication for coming to the hospital is () (multiple choice)

A Chest pain is marked, lasts for several minutes, and recurs. Pain or discomfort in other parts of the upper body.

B The chest pain was obvious and lasted for several minutes, which was not relieved after taking nitroglycerin.

C. Chest discomfort, sudden cold sweat, nausea or dizziness, accompanied by stool feeling.
